# Contrast-enhanced sonography as a novel tool for assessment of vascular malformations

**DOI:** 10.1186/2040-2384-2-25

**Published:** 2010-11-22

**Authors:** Yukiko Oe, Lauren Orr, Sherelle Laifer-Narin, Eiichi Hyodo, Agnes Koczo, Shunichi Homma, Jessica Kandel, Philip Meyers

**Affiliations:** 1 Department of Cardiology, Columbia University, New York, NY, USA; 2 College of Physicians & Surgeons, Columbia University, New York, NY, USA; 3 Department of Radiology, Columbia University, New York, NY, USA; 4 Division of Pediatric Surgery, Columbia University, New York, NY, USA; 5 Department of Neurological Surgery, Columbia University, New York, NY, USA

## Abstract

**Background:**

Vascular malformations with arteriovenous shunt components can cause significant disability, chronic pain, and functional impairment. Effective treatment may require serial procedures, yet an imaging modality optimized to control cost and reduce radiation exposure in this predominantly pediatric population has not yet been identified.

**Methods and Results:**

We describe the use of contrast-enhanced sonography as a novel tool to define vascular anatomy and localize arteriovenous shunting in a young patient with a symptomatic vascular malformation.

**Conclusions:**

This method may effectively reduce radiation exposure and cost, and additionally provide unique information about arteriovenous shunting, offering a novel imaging application for patients with these conditions.

## Background

Vascular malformations (VM) are congenital lesions with diverse clinical manifestations. In contrast to hemangiomas, a separate category of vascular tumors which tend to involute spontaneously, VM often grow in proportion with the child but may expand at an accelerated pace [1]. VM can occur throughout the body, involve multiple vessel types, and may be localized or extensive [2]. Although criteria have been developed for classifying vascular malformations according to flow velocity and vessel type [3], diagnosis of these heterogeneous lesions remains challenging.

While these abnormalities may be asymptomatic, VM become clinically important when associated with disfigurement, functional impairment, pain, infection, and serious bleeding [4,5]. These patients require treatment, which varies according to the specific lesion, its location, functional impairment, and goals of therapy. Assembly of a multi-disciplinary team is advantageous when planning therapy. Further, precise characterization of problematic lesions is critically important both in designing the approach and understanding, likely outcomes (such as the potential need for multiple treatments). For example, malformations with arteriovenous shunting may require embolization [1,6], whereas sclerotherapy is the treatment of choice for venous and macrocystic lymphatic malformations [7,8]. Among venous malformations treated with sclerotherapy, smaller well-defined lesions have been associated with better outcomes than larger ill-defined lesions [6].

Although VM can be partly characterized on the basis of history and physical examination, imaging is commonly required for better identification of abnormal structures in patients with atypical presentations, and to assess the extent of a lesion before and after treatment[9]. Magnetic resonance (MR) imaging is widely considered to be a leading modality for visualizing malformations in relationship to adjacent structures [10,11]. However, the information provided by MR imaging is more sensitive than it is specific [10,12]. Moreover, MR is difficult to use when guiding interventions in real time, and is time- and cost-intensive, frequently requiring sedation or anesthesia for adequate imaging in children. Conversely, ultrasound (US) imaging with and without color Doppler is frequently used in initial screenings because of its availability, safety, relatively low cost, and ability to display flow velocity and to differentiate between types of vascular lesions in real time. However, color Doppler imaging is limited in its ability to detect very small vessels with low or erratic flow [9].

The use of microbubble contrast can improve diagnostic accuracy for echocardiography, and has been used to enhance parenchymal tumor evaluation in clinical and research settings [13,14]. Contrast-enhanced ultrasound (CEUS) enables visualization of vascular architecture in real time, without exposing the patient to ionizing radiation; this may be particularly advantageous to populations in whom such exposure is of concern (such as children) or in whom serial examinations may be necessary (such as patients with vascular malformations). These features suggest that CEUS may be useful to supplement or even replace MRI or conventional angiography in some instances. We report the first case in which CEUS has been used to evaluate a vascular malformation.

## Methods

**Figure 1:**
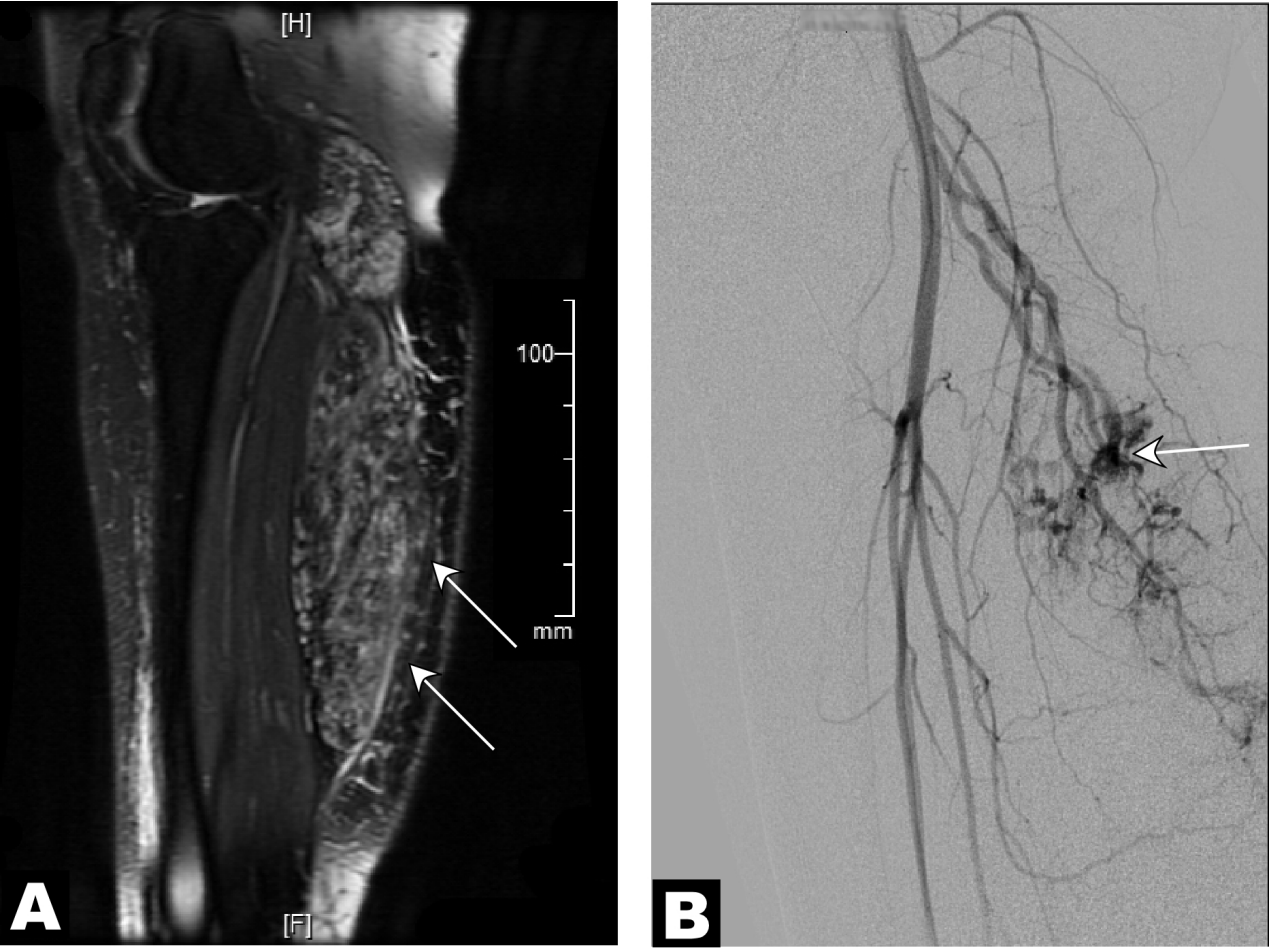
**MR and angiographic studies suggestive of multiple arteriovenous shunts supplying a venous malformation**. A 26-year-old female with a painful vascular malformation of the right lower leg with a clinically obvious venous component was evaluated for arteriovenous shunting. (A) Parasagittal T2-weighted MR image with intravenous gadolinium contrast demonstrated an infiltrative vascular malformation in the right gastrocnemius (*arrows*). (B) Digital subtraction angiogram of the right leg in a lateral projection during the arterial phase revealed arteriovenous shunting (*arrow*) within the vascular malformation, supplied by sural branches of the right popliteal artery.

A 26-year-old woman with a mass in her right calf since the age of 7 presented with continuous pain and swelling in the area. Initial MR imaging demonstrated a vascular malformation (Figure 1). Arteriovenous shunting signifying some component of an arteriovenous malformation was suspected based on the presenting complaints and clinical characteristics of the tense, swollen lesion. Diagnostic arteriography showed a soft tissue malformation with three regions of arteriovenous shunting identified, supplied predominantly by sural branches of the right popliteal artery. Sonography with contrast-enhanced and conventional two-dimensional (2D) color and pulsed Doppler was performed to obtain additional arteriovenous shunt information prior to therapeutic embolization.

Sonographic imaging was performed initially using a Phillips iE33 ultrasound machine with a 2D transducer (S5-1,1. 1.7 - 3.4 MHz). The microbubble contrast agent (Definity™: Lantheus Medical, Billerica, MA) was pre-activated for 45 seconds using a standard diffusion device (Vialmix, Boston Myers Squibb Medical Image. Inc). 1.5 ml Definity was admixed with 9 ml normal saline prior to administration. Areas of arteriovenous shunting overlying her right gastrocnemius muscle, initially visualized at catheter angiography, were scanned with a 2D transducer from the level of the popliteal fossa through the level of the Achilles tendon. Pulsed Doppler interrogation was performed at each specific arteriovenous shunt. After visualizing the whole mass, the transducer was re-positioned at each shunting area using color Doppler imaging. Next, one ml of diluted contrast agent was slowly infused intravenously (1 ml/min). A second one ml dose was administered after 20 minutes. Images were acquired at a low mechanical index (MI 0.31) using the 2D transducer. The contralateral (left) lower leg was scanned as a control study, also using the 2D transducer.

## Results

**Figure 2:**
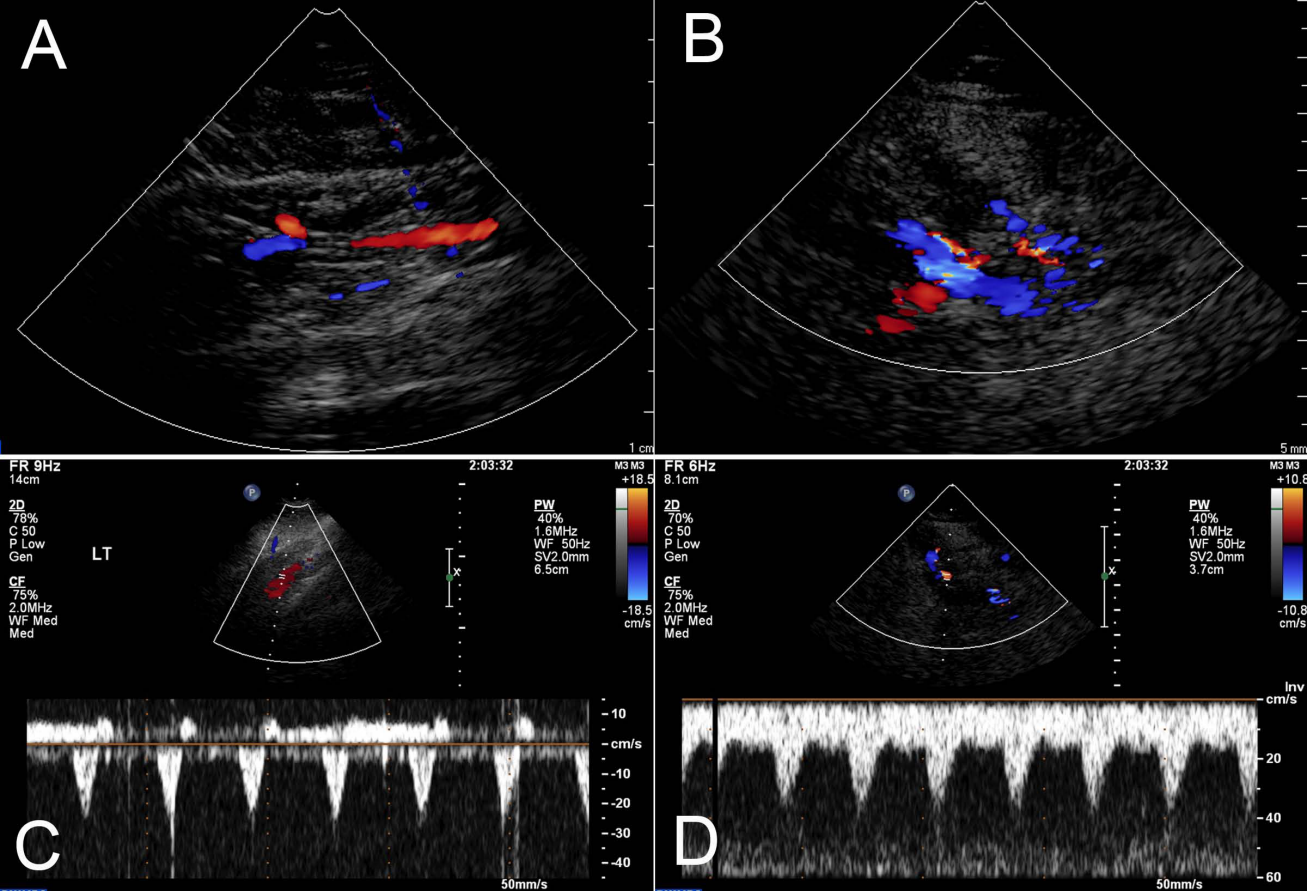
**Contrast-enhanced and color Doppler ultrasound studies**. Two-dimensional ultrasound images were obtained at the level of 3-5 cm below the popliteal fossa (PF). The color Doppler studies demonstrated no abnormal vasculature in the same location on the unaffected leg (A), but multiple tortuous vessel structures with pulsatile flow compatible with venous malformation fed by arteriovenous shunts (B). Pulse Doppler studies identified three specific foci of arteriovenous shunts characterized by low-resistance flow, composed of a normal venous waveform with superimposed arterial pulsatile flow (C). The same studies in the unaffected leg demonstrated a normal arterial wave form (D).

Two-dimensional and color Doppler sonography revealed a multilocular low-flow vascular structure compatible with a venous malformation. Echogenicity and flow signals were greatly enhanced following contrast administration (video clips, Additional Files 1 and 2). However, no such abnormalities were present in the unaffected, contralateral leg. Pulsed Doppler studies localized three specific foci of arteriovenous shunting 3-5 cm below the popliteal fossa (PF) characterized by low-resistance flow, detected as a normal venous waveform with superimposed arterial pulsatile flow (Figure 2).

As a result of the information obtained from this and her prior diagnostic imaging, the patient was treated via a transarterial approach, rather than rather than employing a direct percutaneous approach indicated for less complicated venous malformations [6]. One feeding artery was embolized with acrylic glue (Trufil™ NBCA: Codman Cordis, Rayhnam, MA) and three others were sclerosed with absolute ethanol. Shunts were not detected by post-procedure contrast-enhanced ultrasound, although this study was technically limited and therefore cannot be directly compared with pretreatment images.

## Discussion

Vascular malformations can be a source of significant disability. Appropriate intervention requires accurate characterization of malformation anatomy and vascular architecture. In particular, while identification of arteriovenous shunts is critical to control painful symptoms, techniques to identify shunts can involve repeated exposure to ionizing radiation, contrast, and/or time-intensive and expensive modalities. While sonography with color Doppler is inexpensive, widely available, and safe, it has not been as useful for smaller vessels with low-level perfusion [9]. Of 51 venous malformations evaluated by Trop et al [12], flow in eight malformations could not be detected. The development of sonographic contrast (microbubbles) has the potential to enhance low-flow sonographic echogenicity and Doppler signal, extending the utility of this modality in patients with vascular malformations. In particular, CEUS may offer improved detection of small arteriovenous shunts, as present in this patient with pain and edema in the affected leg. Arteriovenous shunting is important to identify and localize prior to treatment, since it may alter the type of intervention required (e.g. may necessitate embolization instead of or as an adjunct to sclerotherapy).

In this case, arteriovenous shunting was suspected due to the pain and progressive swelling associated with the lesion. Multiple arteriovenous shunts were indeed visualized using contrast-enhanced ultrasound, including at least one region that had not been clearly apparent on either MRI or conventional diagnostic angiography, and were confirmed at the time of therapeutic angiography. As a result of the additional information obtained from this diagnostic imaging, the patient was treated transarterially, with combined embolization of a feeding artery and sclerosis of three communicating venous channels.

Malformations with substantial venous components often require staged treatments[2,7]. Multiple treatments are often necessary for several reasons. First, venous malformations may recanalize [7,10]. This limitation cannot be overcome by increased dosing of sclerosant, since these agents (such as ethanol) act by damaging cells and, at higher doses, can cause skin necrosis, hemolysis, and peripheral nerve injury [11,15]. Furthermore, in venous malformations involving arteriovenous shunt components, new or previously inapparent shunts may cause recurrence of pain or swelling. Because the majority of patients presenting with vascular malformations are children, an optimal imaging strategy would maximize detection of arteriovenous shunts, while minimizing radiation exposure, iodinated contrast load needed for conventional angiography, requirement for sedation or anesthesia, and cost.

An additional advantage of an optimal imaging strategy might include the real-time ability to guide intervention. Lewin et al [6] added trace amounts of MR contrast to ethanolamine and were able to safely perform MR imaging-guided sclerotherapy. Similarly, microbubble contrast could be added to a sclerosing agent in order to guide treatment by sonography, which would reduce the radiation load to patients and healthcare workers. Intriguingly, microbubbles have proven to be highly customizable. For example, microbubbles can be engineered to bind specific epitopes in vasculature, widely demonstrated in animal models and in development for humans [16]. This capability could be very useful in treatment of patients whose vascular anomalies affect specific subsets of vessels, or which stem from particular mutations.

## Conclusions

We report the use of CEUS in a young patient with a complex, disabling vascular malformation. Our experience suggests that this modality has the potential to supplement or replace current vascular imaging modalities. Further, CEUS may have particular utility in detection of small arteriovenous shunts that are more difficult to detect with conventional sonography or MR imaging. The plasticity of microbubbles as a contrast platform may offer particular advantages for future development of novel targeted intravascular therapies.

## Competing interests

The authors declare that they have no competing interests.

## Authors' contributions

YO, EK, and AH carried out the ultrasound studies, and YO participated in their analysis, and edited the relevant portions of the manuscript. LO participated in the ultrasound studies and the angiographic treatment, and drafted the introduction to the manuscript. YO and SLN participated in planning contrast-enhanced ultrasound, and performed the post-treatment study. SH participated in the planning the contrast-enhanced ultrasound approach and technique. JK and PM conceived of the study, participated in its design and coordination, composed the conclusions, and edited the manuscript. PM additionally performed the angiographic treatment for this patient. All authors read and approved the final manuscript.

## Supplementary Material

Additional file 1 **Video clip 1: Area of vascular anomaly with swelling at time 0 of contrast administration**. Contrast enhanced ultrasound studies permit detection of small vessels not visualized on conventional ultrasound. This technique allows identification of smaller anomalous vessels, which were not detectable by non-contrast B mode sonography, as in this pre-contrast video clip. Such vasculature may contribute to the pathophysiology of the anomaly, and thus identifying contributory vessels may be useful in planning interventions. Click here for file

Additional file 2 **Video clip 2: Area of vascular anomaly two minutes after contrast administration**. Vasculature can be detected in the region clinically involved by malformation after contrast administration. Click here for file

## References

[B1] Dubois J Garel L Imaging and therapeutic approach of hemangiomas and vascular malformations in the pediatric age group Pediatr Radiol 1999 29 879 893 10.1007/s002470050718 10602864

[B2] Garzon MC Huang JT Enjolras O Frieden IJ Vascular malformations: Part I J Am Acad Dermatol 2007 56 353 370 quiz 371-354 10.1016/j.jaad.2006.05.069 17317485

[B3] Mulliken JB Glowacki J Hemangiomas and vascular malformations in infants and children: a classification based on endothelial characteristics Plast Reconstr Surg 1982 69 412 422 10.1097/00006534-198203000-00002 7063565

[B4] Mathes EF Haggstrom AN Dowd C Hoffman WY Frieden IJ Clinical characteristics and management of vascular anomalies: findings of a multidisciplinary vascular anomalies clinic Arch Dermatol 2004 140 979 983 10.1001/archderm.140.8.979 15313815

[B5] Elsayes KM Menias CO Dillman JR Platt JF Willatt JM Heiken JP Vascular malformation and hemangiomatosis syndromes: spectrum of imaging manifestations AJR Am J Roentgenol 2008 190 1291 1299 10.2214/AJR.07.2779 18430846

[B6] Lewin JS Merkle EM Duerk JL Tarr RW Low-flow vascular malformations in the head and neck: safety and feasibility of MR imaging-guided percutaneous sclerotherapy--preliminary experience with 14 procedures in three patients Radiology 1999 211 566 570 10228544 10.1148/radiology.211.2.r99ma09566

[B7] Marler JJ Mulliken JB Current management of hemangiomas and vascular malformations Clin Plast Surg 2005 32 99 116 ix 10.1016/j.cps.2004.10.001 15636768

[B8] Konez O Burrows PE An appropriate diagnostic workup for suspected vascular birthmarks Cleve Clin J Med 2004 71 505 510 10.3949/ccjm.71.6.505 15242306

[B9] Paltiel HJ Burrows PE Kozakewich HP Zurakowski D Mulliken JB Soft-tissue vascular anomalies: utility of US for diagnosis Radiology 2000 214 747 754 10715041 10.1148/radiology.214.3.r00mr21747

[B10] Dubois J Soulez G Oliva VL Berthiaume MJ Lapierre C Therasse E Soft-tissue venous malformations in adult patients: imaging and therapeutic issues Radiographics 2001 21 1519 1531 11706222 10.1148/radiographics.21.6.g01nv031519

[B11] Hein KD Mulliken JB Kozakewich HP Upton J Burrows PE Venous malformations of skeletal muscle Plast Reconstr Surg 2002 110 1625 1635 10.1097/00006534-200212000-00001 12447041

[B12] Trop I Dubois J Guibaud L Grignon A Patriquin H McCuaig C Garel LA Soft-tissue venous malformations in pediatric and young adult patients: diagnosis with Doppler US Radiology 1999 212 841 845 10478255 10.1148/radiology.212.3.r99au11841

[B13] Weyman AE Future directions in echocardiography Rev Cardiovasc Med 2009 10 4 13 10.2459/JCM.0b013e328313e8bb 19367227

[B14] von Herbay A Vogt C Willers R Haussinger D Real-time imaging with the sonographic contrast agent SonoVue: differentiation between benign and malignant hepatic lesions J Ultrasound Med 2004 23 1557 1568 15557299 10.7863/jum.2004.23.12.1557

[B15] Puig S Aref H Chigot V Bonin B Brunelle F Classification of venous malformations in children and implications for sclerotherapy Pediatr Radiol 2003 33 99 103 12557065 10.1007/s00247-002-0838-9

[B16] Pochon S Tardy I Bussat P Bettinger T Brochot J von Wronski M Passantino L Schneider M BR55: a lipopeptide-based VEGFR2-targeted ultrasound contrast agent for molecular imaging of angiogenesis Invest Radiol 45 89 95 10.1097/RLI.0b013e3181c5927c 20027118

